# The regulatory role of alternative splicing in inflammatory bowel disease

**DOI:** 10.3389/fimmu.2023.1095267

**Published:** 2023-04-21

**Authors:** Jianli Zhou, Qiao Zhang, Yuzhen Zhao, Yuchen Song, Yanan Leng, Moxian Chen, Shaoming Zhou, Zhaoxia Wang

**Affiliations:** ^1^ Department of Gastroenterology, Shenzhen Children’s Hospital, Shenzhen, Guangdong, China; ^2^ Co-Innovation Center for Sustainable Forestry in Southern China and Key Laboratory of National Forestry and Grassland Administration on Subtropical Forest Biodiversity Conservation, College of Biology and the Environment, Nanjing Forestry University, Nanjing, China; ^3^ Clinical Laboratory, Shenzhen Children’s Hospital, Shenzhen, Guangdong, China

**Keywords:** alternative splicing, Crohn’s disease, inflammatory bowel disease, splicing factor, splicing mutation, transcriptome, ulcerative colitis

## Abstract

Inflammatory bowel disease (IBD) mainly includes Crohn’s disease and ulcerative colitis. These diseases have a progressive course of chronic relapse and remission and affect a large number of children and adults worldwide. The burden of IBD is rising worldwide, with levels and trends varying greatly in countries and regions. Like most chronic diseases, the costs associated with IBD are high, including hospitalizations, outpatient and emergency visits, surgeries, and pharmacotherapies. However, there is no radical cure for it yet, and its therapeutic targets still need further study. Currently, the pathogenesis of IBD remains unclear. It is generally assumed that the occurrence and development of IBD are related to the environmental factors, gut microbiota, immune imbalance, and genetic susceptibility. Alternative splicing contributes to a various diseases, such as spinal muscular atrophy, liver diseases, and cancers. In the past, it has been reported that alternative splicing events, splicing factors, and splicing mutations were associated with IBD, but there were no reports on the practical application for clinical diagnosis and treatment of IBD using splicing-related methods. Therefore, this article reviews research progress on alternative splicing events, splicing factors, and splicing mutations associated with IBD.

## 1 Introduction

Inflammatory bowel disease (IBD) includes ulcerative colitis (UC), Crohn’s disease (CD), and indeterminate colitis. These diseases have a progressive course of chronic relapse and remission and affect a large number of children and adults worldwide (1). IBD can occur at any age but is most common among adolescents and young adults (2). According to age, IBD is divided into adult-onset IBD and pediatric-onset IBD. The latter includes neonatal IBD, infantile-onset IBD, very early–onset IBD (VEO-IBD; diagnosed before 6 years old), and early-onset IBD (EO-IBD; diagnosed before 10 years old) (2). The prevalence of IBD is approximately 1% in developed countries, leading to an increasing prevalence while the incidence and prevalence are also rising in developing countries (2). This rise in prevalence and increased incidence will have important global health and economic effects (2). As there is no cure for IBD, the costs are high with a mean cost of $26,555 in the first year after initial diagnosis, including hospitalizations, outpatient and emergency visits, surgeries, and pharmacotherapies (3). Therefore, there is a need to develop effective treatments for IBD.

**Figure 1:**
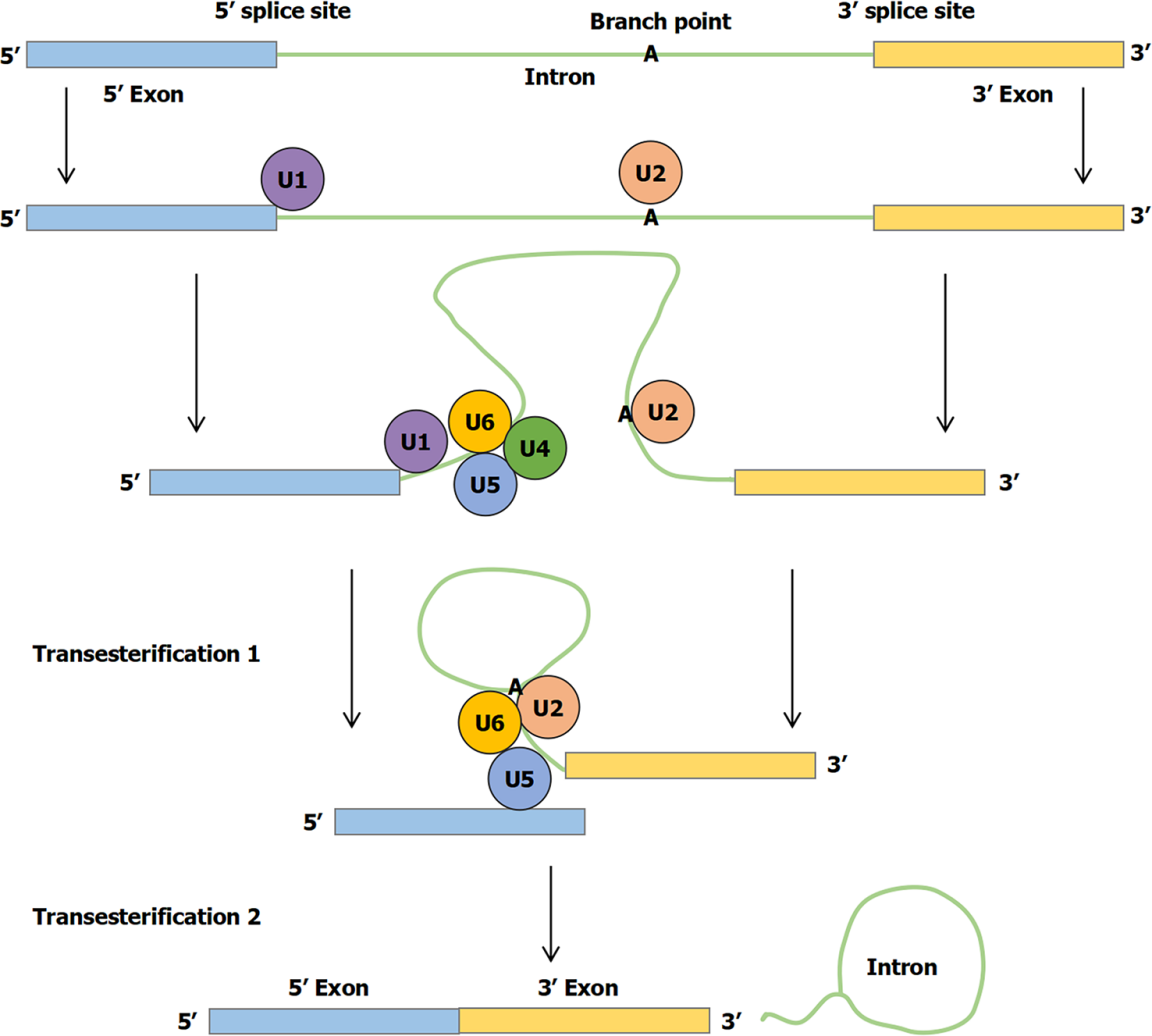
The mechanism of RNA splicing. First, a branch point A nucleotide located near the 3′ splice site of the intron sequence attacks the 5′ splice site and clefts it. The 5′ end of the intronic sequence cleavage is thus covalently linked to this A nucleotide, forming the branched nucleotide. Second, the 3′-OH end of the first exon sequence created in the first step is added to the beginning of the second exon sequence to cleave the RNA molecule at the 3′ splice site. Thus, the two exonic sequences are linked to each other and the intronic sequence is released as a lasso.

Currently, the pathogenesis of IBD remains unclear. It is generally assumed that the occurrence and development of IBD are related to the environmental factors, gut microbiota, immune imbalance, and genetic susceptibility, which can impact a weakened gut barrier on the microbiota, leading to inappropriate intestinal immune activation (4). The genetics of IBD have benefited substantially from studying common variants in large cohorts of patients with good phenotypes and from studying rare variants in cases associated with Mendelian inheritance (5). Before being translated into protein, primary mRNA must be modified and edited. During normal splicing, a special protein and RNA complex, called the spliceosome, attaches to the mRNA. Specific RNA transcript product regions (introns) are excised, whereas flanking sequences (exons) are spliced together. This process fundamentally changes the information content of RNA transcripts directly affecting translation of genetic information into proteins. Regulation of splicing thus represents a critical step in gene expression occurring in the nucleus (**Figure 1**) (6). First, a branch point A nucleotide located near the 3′ splice site of the intron sequence attacks the 5′ splice site and cleaves it. The 5′ end of the intronic sequence cut is thus covalently linked to this A nucleotide, forming a branched nucleotide. Second, the 3′-OH end of the first exon sequence created in the first step is added to the beginning of the second exon sequence to cleave the RNA molecule at the 3′ splice site. Thus, two exonic sequences are linked to each other, and the intronic sequence is released as a lasso (6). At present, alternative splicing has been reported in many diseases, including spinal muscular atrophy (SMA), liver diseases, and cancers (7–9). For example, Nusinersen targets intronic splice silencer N1 to modulate splicing of survival motor neuron 2 (SMN2) exon 7, resulting in increased production of full-length SMN for SMA therapy (7).

On the basis of the Web of Science core collection database, approximately 6–19 articles have been published in this category each year for the last decade (**Figure S1A**). Hot keywords include IBD, ulcerative colitis, Crohn’s disease, expression, activation, genome-wide association, pathogenesis, splicing variant and splice site mutation (**Figure S1B**). Some keywords are related together, such as ulcerative colitis, splicing variant, and colorectal cancer, whereas the remaining keywords are related in the other three parts (**Figure S1C**). In this review, we will summarize the research on alternative splicing events, splicing factors, and splicing mutations in IBD and highlight their role in diseases.

## 2 Alternative splicing in IBD

Alternative splicing is associated with the pathogenesis of IBD and may serve as a potential therapeutic target for clinical therapy. Previous studies have reported an association between alternative splicing and the pathogenesis of CD and UC (10, 11). Li’s team demonstrated a novel potential pathophysiological mechanism for alternative splicing associated with CD, presenting the first alternative splicing landscape in patients with CD, suggesting that drugs targeting alternative splicing-related genes [monoamine oxidase A (*MAOA*), signal transducer and activator of transcription 3 (*STAT3*), and interferon regulatory factor 1 (*IRF1*)] and splicing factors can be used for screening and treatment of patients with CD (10). They also presented a study of alternative splicing events in patients with UC, where exon deletion and substitution of the first exon were the two most significantly altered events (11). Immune response–related pathways were significantly enriched in UC-associated alternative splicing events, suggesting a strong association between dysregulation of alternative splicing and pathogenesis of UC. Moreover, histone deacetylase 6 (*HDAC6*) and lipase A (*LIPA*) had significant alternative splicing events in patients with UC involved in immune responses and inflammation, indicating that dysregulated alternative splicing of *HDAC6* or *LIPA* may be a potential therapeutic target for patients with UC. In this section, we summarize studies on alternative splicing in adults and children with IBD (**Table 1**).

**Table 1:** Studies reporting alternative splicing in IBD of children and adults.

Study (Ref.)	Methods	Potential function	Disease	Adults/children
Häsler et al. (12)	The occurrence and differential expression of 149 splicing factors in patients with IBD.	Pathogenesis	IBD	Adults
Häsler et al. (13)	The microbiome, transcriptome, and splicing analysis were performed in patients with IBD by using next-generation sequencing.	Pathogenesis	IBD	Adults
Rankin et al. (14)	Identify specific splicing variants in interferon gamma-antisense 1 (IFNG-AS1) that were associated with differing degrees of patients with IBD.	Pathogenesis	IBD	Adults
Berger et al. (15)	Transcriptome profiles were assessed by RNA-seq of ileal and rectal tissues from 34 pediatric or young adult with IBD.	Pathogenesis	IBD	Adults and children
Díez-Obrero et al. (16)	The gene expression and alternative splicing of 445 healthy individuals showed that single-nucleotide polymorphisms (SNPs) were enriched in disease-associated loci, including IBD.	Pathogenesis	IBD	Adults
Iacomino et al. (17)	Evaluated the expression of Treg, Th1, and Th17 cytokines, and the splicing of X-box binding protein-1 (XBP1) mRNA in patients with IBD.	Pathogenesis	IBD	Adults
Bootz et al. (18)	Alternating spliced EDA domain of fibronectin was expressed in patients with IBD.	Therapeutic target	IBD	Adults
Pai et al. (19)	In patients with IBD, the expression levels of the splice variants myosin light chain kinase 1 (MLCK1) and MLCK2 were significantly increased.	Therapeutic target	IBD	Adults
Derer et al. (20)	In patients with IBD, glycoprotein 2 splicing variant 4 (GP2#4) was expressed on intestinal M or L cells with an elevated expression pattern.	Therapeutic target	IBD	Adults
Li et al. (21)	Increased IGF-IEc expression and mechano-growth factor production in fibrostenotic CD.	Pathogenesis	CD	Adults
Li et al. (10)	Reanalysis of mRNA-seq data and identified alternative splicing events in patients with CD.	Pathogenesis	CD	Adults
Manousou et al. (22)	The mRNA expression of C-X-C motif chemokine Receptor 3 (CXCR3) splice variant in CD3+ peripheral blood lymphocytes (PBLs) was obvious in patients with CD.	Pathogenesis	CD	Adults
Gong et al. (23)	To investigate the correlation between spliceosomal associated protein 130 (SAP130) level and CD severity, and the clinical efficacy of CD remission induced by exclusive enteral nutrition (EEN).	Biomarker	CD	Adults
Towers et al. (24)	IL-18 involved in the alternative splicing of human glucocorticoid receptor (hGR) mRNA in patients with CD.	Predictor of glucocorticoid resistance	CD	Adults
Mailer et al. (25)	Alternative splicing of forkhead box P3 (FOXP3) in patients with CD.	Therapeutic target	CD	Adults
Wittig et al. (26)	Elevated levels of the CD44v7 (one of the isoforms alternatively spliced exon of CD44) ligand osteopontin in CD induced IL-6 in human monocytes, a cytokine also increased in these patients.	Pathogenesis and therapeutic target	CD	Adults
Tang et al. (27)	The splice forms of cylindromatosis lysine 63 deubiquitinase (sCYLD) mediated ubiquitination and nuclear translocation of SMAD family member 7 (SMAD7).	Therapeutic target	CD	Adults
Low et al. (28)	The results showed that many genes exhibited different kinds of alternative splicing in UC.	Pathogenesis	UC	Adults
Muise et al. (29)	Three SNPs including rs886936, rs17130, and rs8100586, associated with novel splicing that removes the third immunoglobulin-like domain (exon 9) from the human protein tyrosine phosphatase receptor type S (PTPRS) gene, were associated with UC.	Pathogenesis	UC	Adults
Li et al. (30)	The splicing of XBP1 in patients with UC was greater in costimulation than in single stimulation.	Pathogenesis	UC	Adults
Xiu et al. (31)	Differentially expressed genes (DEGs) analysis was performed using the microarray dataset GSE87473 in 19 children and 87 adult UC from the Gene expression Omnibus.	Biomarker	UC	Adults and children
Li et al. (11)	Genome-wide analysis of alternative splicing characteristics in patients with UC.	Pathogenesis and therapeutic target	UC	Adults
Oh et al. (32)	A synonymous variants p.T179T of interleukin-10 receptor subunit alpha (*IL10RA*) affected RNA splicing by influencing exon skipping and out-of-frame fusion of exons 3 and 5 in pediatric patients with CD.	Pathogenesis	CD	Children

IBD, inflammatory bowel disease; CD, Crohn’s disease; UC, ulcerative colitis.

### 2.1 Alternative splicing in adults with IBD

Alternative splicing is associated with the pathogenesis of IBD and may serve as a potential therapeutic target for IBD in adults (12–20). One study showed that the regulation of seven splicing factors (*HNRPAB*, *DUSP11*, *HNRPH3*, *SF3B14*, *SFPQ*, *SLU7*, and *SFR2IP*) is IBD specific (12). The expression patterns of these seven splicing factors did not share a regulatory pattern with the intron retention patterns of *FGD2*, *PARC*, and *IER3*, indicating that splicing factors have a potential influence on subsequent regulated intron retention in IBD pathogenesis. Years later, their team revealed a close interaction between the microbiome, gene regulation, and splicing architecture in the pathogenesis of IBD (13). Another study found that the second splicing variant of interferon gamma-antisense 1 (*IFNG-AS1*) is positively correlated with the severity of IBD, and a single-nucleotide polymorphism (SNP) in *IFNG-AS1*, rs7134599, is associated with the pathophysiology of IBD (14). Furthermore, the researchers found that two patients with IBD have 284 exons with significantly different splicing rates at exons, including *CEACAM1*, an established IBD risk gene, suggesting that splicing analysis derived from RNA-seq experiments may play an important role in identifying pathogenesis of IBD (15). Recently, a study provided gene expression and alternative splicing data for 445 healthy individuals showing that SNPs are enriched in disease-associated loci, including IBD, resulting in provision of the Colon Transcriptome Explorer web application (16). Another study evaluated mRNA splicing for transcription factor X-box binding protein-1 (*XBP1*) by quantitative reverse transcriptase real-time PCR to measure relative expression of Th1, Th17, and Treg cytokines, finding that mRNA splicing for transcription factor *XBP1* may be involved in immunopathogenesis of IBD (17).

In addition to pathogenesis, alternative splicing may serve as a potential therapeutic target for IBD (18–20). One study showed that the alternatively spliced fibronectin EDA domain is expressed in patients with IBD, whereas it is barely detectable in most normal adult tissues (18). Thus, they identified the alternatively spliced EDA domain of fibronectin as a target for drug delivery applications in IBD. Moreover, different splice variants of myosin light chain kinase (*MLCK*) (including *MLCK1* and *MLCK2*) could regulate paracellular and extracellular permeability in IBD (19). First-line therapy could be developed to inhibit tumor necrosis factor–like cytokine 1A–mediated MLCK2-dependent bacterial endocytosis, which may serve as a therapeutic target for patients with IBD. Autoantibodies against different splice variants of glycoprotein 2 (GP2) frequently occur in patients with IBD (20). The last study showed that autoimmunity against gut-expressed GP2 splicing variant 4 (GP2#4) resulted in enhanced adhesion of flagellar bacteria to intestinal epithelium and thus may drive the pathophysiology of IBD (20). Blocking or depleting GP2#4-directed autoantibodies with anti-idiotypic antibodies may be a new therapeutic target for IBD.

### 2.2 Alternative splicing in adults with CD

Alternative splicing is associated with the pathogenesis of CD and may serve as a potential biomarker, predictor of glucocorticoid (GC) resistance, and therapeutic target for CD in adults (10, 21–26, 33). The alternative splicing of insulin-like growth factor 1 (*igf1*) gene includes IGF-IEa and IGF-IEc (21). Researchers found increased IGF-IEc expression and mechano-growth factor production in patients with fibrostenotic CD, indicating that alternative splicing of *igf1* may be related to pathogenesis of fibrostenotic CD (21). Li et al. reanalyzed mRNA-seq data providing a landscape of alternative splicing events in CD, suggesting that alternative splicing disorder may be a mechanism in pathogenesis of CD (10). Another study showed that mRNA expression of C-X-C motif chemokine receptor 3 (*CXCR3*) splice variants is detected in CD3+ peripheral blood lymphocytes (PBL) of patients with CD, indicating that *CXCR3* splicing may play a significant role in pathogenesis of CD (22).

Previous studies found that serum spliceosome-associated protein 130 (SAP130) levels in patients with active CD are significantly higher than in patients with remission CD and controls and changed with different disease activities being significantly correlated with disease severity (23). Furthermore, serum SAP130 level of patients with active CD decreased after 8 weeks of exclusive enteral nutrition (EEN), demonstrating that SAP130 may be a potential biomarker for assessing the severity and clinical efficacy of EEN in adults with CD. A study showed that amount of human GC receptor (hGR) β-mRNA is significantly higher in patients with GC-resistant CD in active stage (24). IL-18 content was directly correlated with hGRβ-mRNA content in patients with active CD with GC resistance, indicating that IL-18 may be involved in the alternative splicing of hGR in patients with CD. It seemed that alternative splicing of hGR may be a predictor of GC resistance in patients with CD.

**Figure 2:**
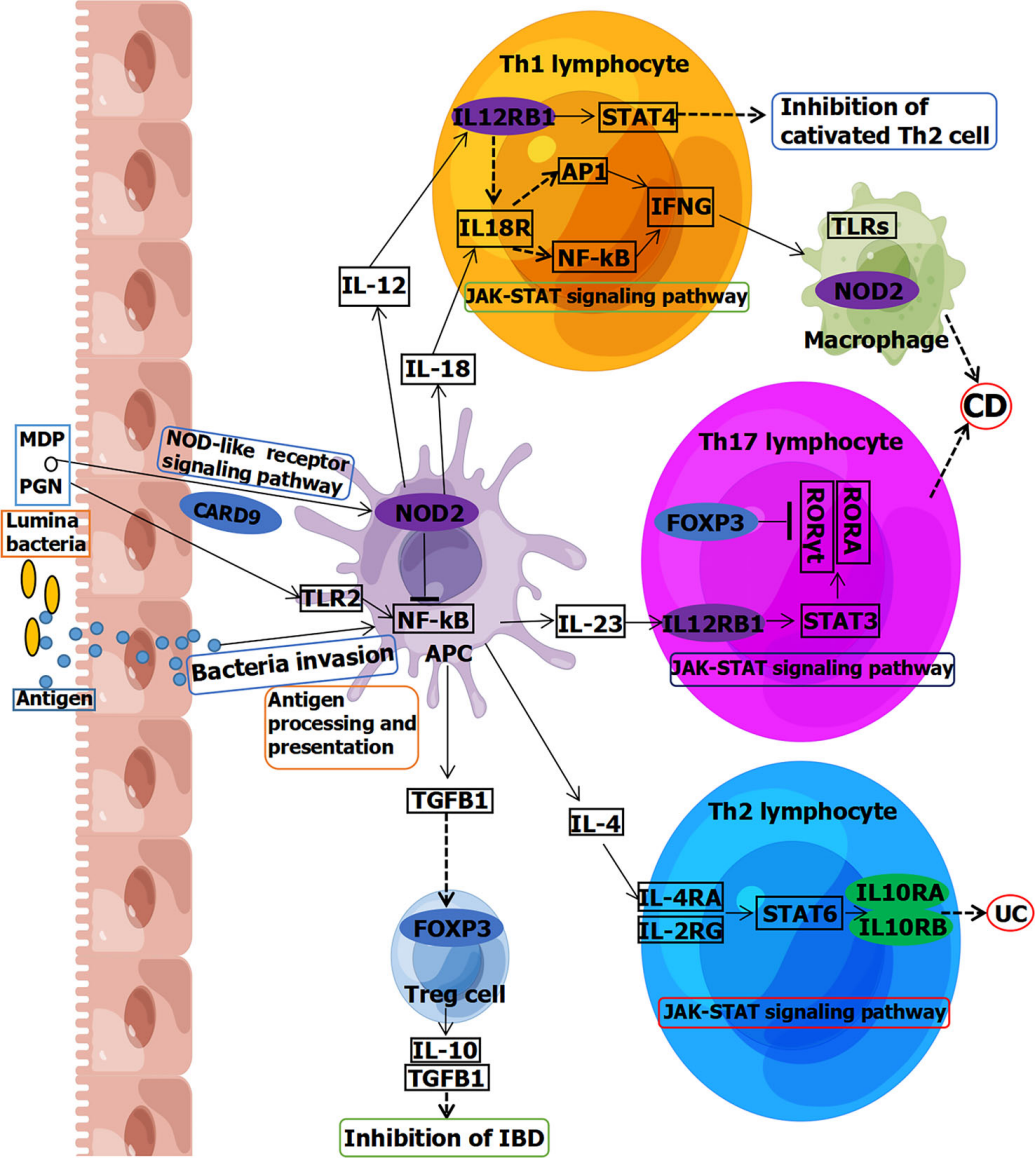
Partial signaling pathways of IBD associated with splicing, including CD and UC. Genes circled in ovals are involved in splicing. Blue is reported in adults. Green is reported in children. Purple is reported both in children and adults. MDP, muramyldipeptide; PGN, peptidoglycans; TLR, Toll-like receptor; NF-kB, nuclear factor–kappa B; APC, antigen-presenting cell; IFNG, gamma interferon; STAT, signal transducer and activator of transcription; JAK, Janus kinase; RORγt, retinoic acid–related orphan receptor gamma; RORA, retinoic acid–related orphan receptor alpha; TGFB1, transforming growth factor beta 1; FOXP3, forkhead box P3; CARD9, caspase recruitment domain family member 9; IL12RB1, interleukin-12 receptor subunit beta 1; NOD2, nucleotide-binding oligomerization domain containing 2; IL10RA, interleukin-10 receptor subunit alpha; IL10RB, interleukin-10 receptor subunit beta. This figure was drawn using Figdraw (ID: YYSUS3f0fd).

Moreover, forkhead box P3 (*FOXP3*) is involved in Janus kinase–signal transducer and activator of transcription (JAK-STAT) signaling pathway in Th17 lymphocytes, leading to CD (**Figure 2**) (33). *FOXP3* is also involved in a pathway in Treg cell inhibition of IBD (**Figure 2**). Previous research reported that alternative splicing of *FOXP3* modulated T-cell differentiation, which may be a therapeutic target for patients with CD (25). Levels of CD44v7 (one of the isoforms alternatively spliced exon of CD44) ligand osteopontin were elevated in CD, inducing IL-6 in human monocytes (26). This indicated that CD44v7 may play an important role in pathogenesis of CD and be a novel therapeutic target for CD. Another study showed that the levels of the spliced form of cylindromatosis lysine 63 deubiquitinase (sCYLD) are increased in colon tissues of patients with CD (27). Ubiquitination and nuclear translocation of SMAD family member 7 (SMAD7) were mediated by sCYLD to reduce transforming growth factor β signaling in T cells, indicating that sCYLD-SMAD7 complex may be a therapeutic target for CD.

### 2.3 Alternative splicing in adults with UC

Alternative splicing is also associated with pathogenesis of UC and may serve as a valuable biomarker for clinical diagnosis and therapy in adults (11, 28–31). Alternative splicing events, gene expression, and pathways were altered in patients with UC with long and short disease durations, linking alternative splicing to pathogenesis of UC (28). Receptor protein tyrosine phosphatase sigma (*PTPRS*-encoding PTPσ) knockout mice may spontaneously develop mild colitis (29). Presence of some SNPs was associated with novel splicing that removed a third immunoglobulin-like domain (exon 9) from extracellular portion of PTPσ, potentially altering dimerization or ligand recognition. This indicated that splicing of the *PTPRS* gene may also be related to pathogenesis of UC. Furthermore, splicing of *XBP1* in patients with UC with costimulation was greater than those with single stimulation (30). This indicated that splicing of *XBP1* may contribute to pathogenesis of UC. Moreover, a previous study showed that some genes (*POLR2A*, *CDC42*, *PIK3R1*, *RAC1*, *SRC*, and *MAPK1*) are mainly enriched in intercellular items related to cell junction, cell adhesion, actin cytoskeleton, transmembrane receptor signaling pathways, and intracellular items related to RNA splicing, metabolism, and localization (31). These results suggested that these genes related to splicing may be associated with pathogenesis of adults with UC and serve as diagnostic and therapeutic targets of UC. In addition, abnormal regulation of alternative splicing may play a key role in pathogenesis and serve as a potential therapeutic target of UC (11).

### 2.4 Alternative splicing in children with IBD

Currently, only a few studies have reported on relationship between alternative splicing and IBD in children (15, 31, 32). Researchers found that two patients with IBD have 284 exons with significantly different splicing rates at exons, including *CEACAM1*, an established IBD risk gene (15). This suggested that splicing analysis derived from RNA-seq experiments may play an important role in identifying pathogenesis of IBD. Synonymous variants of *IL10RA* have been reported to affect RNA splicing in pediatric patients with CD, which may be associated with pathogenesis of children with CD (32). Because genes (*POLR2A*, *CDC42*, *PIK3R1*, *RAC1*, *SRC*, and *MAPK1*) were mainly enriched in intercellular items related to cell junction, cell adhesion, actin cytoskeleton, transmembrane receptor signaling pathways, and intracellular items related to RNA splicing, metabolism, and localization, splicing of above genes may be associated with pathogenesis of UC, serving as potential biomarkers for clinical diagnosis and therapy in children (31). Alternative splicing has been studied more in adult IBD than in children, indicating a need for further studies on alternative splicing in pediatric IBD.

## 3 Splicing factors in IBD

There is considerable evidence that diseases are associated with abnormal expression of splicing factors (34–37). Many splicing factors have been reported to be expressed in patients with IBD (**Table 2**) (10–12). Regulatory patterns of seven splicing factors (*HNRPAB*, *DUSP11*, *HNRPH3*, *SF3B14*, *SFPQ*, *SLU7*, and *SFR2IP*) observed in a study were specific to IBD rather than solely dependent on inflammation (12). *SF3B14* and *SFPQ* showed upregulation in patients with CD but not in those with UC, indicating that the splicing factor expression levels of UC and CD are different and play different roles in different disease subtypes, suggesting that a mechanism of this aspect may serve as a diagnostic marker for IBD in the future.

**Table 2:** Studies reporting alterations of RNA splicing factor expression in IBD.

Gene symbol	Description	Potential function	Disease	Adults/children	Ref.
DUSP11	Dual-specificity phosphatase 11	Diagnostic marker	IBD	Adults	(12)
HNRPAB	Heterogeneous nuclear ribonucleoprotein A/B	Diagnostic marker	IBD	Adults	(12)
HNRPH3	Heterogeneous nuclear ribonucleoprotein H3	Diagnostic marker	IBD	Adults	(12)
SLU7	Zn-finger protein	Diagnostic marker	IBD	Adults	(12)
SFR2IP	A splicing factor	Diagnostic marker	IBD	Adults	(12)
SFPQ	Proline- and glutamine-rich	Diagnostic marker	IBD	Adults	(12)
SF3B14	Splicing factor 3B, subunit 14	Diagnostic marker	IBD	Adults	(12)
HSPA1A	Heat shock protein family A (Hsp70) member 1A	Pathogenesis	CD	Adults	(10)
RNF213	Ring finger protein 213	Pathogenesis	CD	Adults	(10)
MOV10	Mov10RISC complex RNA helicase	Pathogenesis	CD	Adults	(10)
HSPA5	Heat shock protein family A (Hsp70) member 5	Pathogenesis	CD, UC	Adults	(10, 11)
LSM10	U7 small nuclear RNA–associated	Pathogenesis	CD	Adults	(10)
ELAVL3	ELAV-like RNA binding protein 3	Pathogenesis	UC	Adults	(11)
ZMAT5	Zinc finger matrin-type 5	Pathogenesis	UC	Adults	(11)
BAG2	BAG cochaperone 2	Pathogenesis	UC	Adults	(11)
TXNL4A	Thioredoxin Like 4A	Pathogenesis	UC	Adults	(11)

Splicing factors have also been reported in two subtypes of IBD (10, 11). One study identified five splicing factors (*HSPA1A*, *RNF213*, *MOV10*, *HSPA5*, and *LSM10*) that are significantly differentially expressed in patients with CD, playing a key role in the regulation of alternative splicing events in patients with CD (10). Another study also identified five splicing factors (*ELAVL3*, *ZMAT5*, *BAG2*, *HSPA5*, and *TXNL4A*) that are significantly differentially expressed in patients with UC, closely related to change in immune response in patients with UC (11). These studies revealed potential splicing factors in mechanism of alternative splicing changes in patients with CD or UC.

## 4 Mutations of splicing or affected splicing in genes associated with IBD

**Figure 3:**
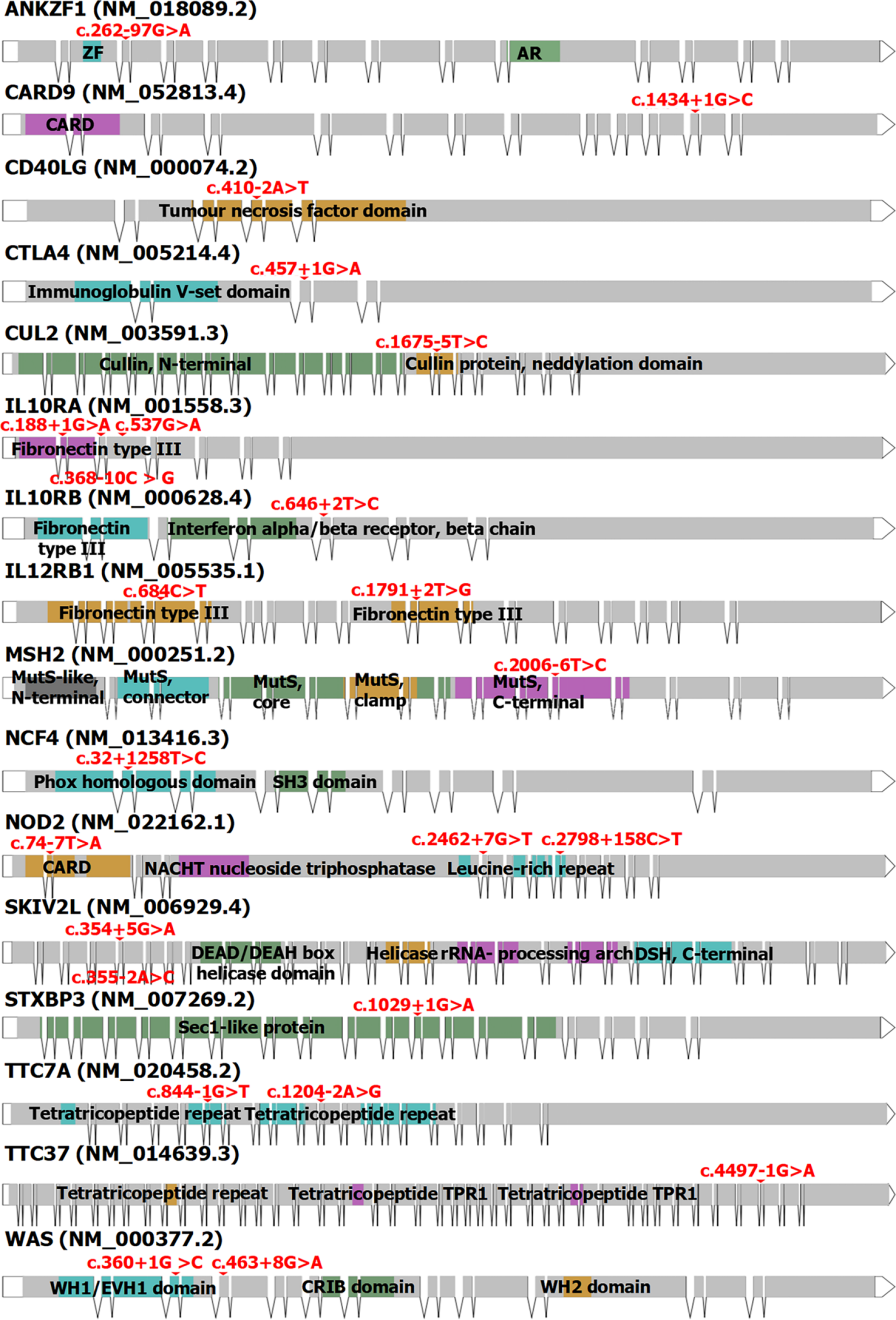
The structure of the 16 genes associated with IBD (including CD and UC). Red: the mutation sites of splicing or resulting in splicing. Blue, green, orange, purple, and black: the domain locations of every gene from Prosite or Pfam. ZF, zinc finger; AR, ankyrin repeat; CARD, caspase recruitment domain; MutS, DNA mismatch repair protein MutS; SH3, Src homology 3; NACHT, the NAIP, CIITA, HET-E, and TP1 domain with NTPase activity; DSH, dynein stalk head; TPR1, tetratricopeptide repeat 1; WH1, Wiskott–Aldrich syndrome protein homology 1; EVH1, enabled vasodilator-stimulated protein homology 1; CRIB, Cdc42 and Rac interactive binding domain; WH2, Wiskott–Aldrich syndrome protein homology 2. The full names of these genes are shown in **Table 3**.

To date, more than 200 genes affecting or associated with IBD have been reported, with a total of more than 600 mutation sites reported in the Human Gene Mutation Database (v2022.6) (38, 39). According to genetic classification, there are currently 31 types of IBD, including IBD1 (OMIM #266600), IBD2 (OMIM #601458), and IBD3 (OMIM #604519) (40). Currently, 24 mutations of splicing or associated with splicing have been reported in 16 genes associated with IBD. Among them, genes associated with splicing mutations in adult IBD include *CARD9*, *CUL2*, *IL12RB1*, *MSH2*, *NCF4*, and *NOD2*. In pediatric IBD, they include *ANKZF1*, *CD40LG*, *CTLA4*, *IL10RA*, *IL10RB*, *IL12RB1*, *NCF4*, *NOD2, SKIV2L*, *STXBP3*, *TTC37*, *TTC7A*, and *WAS* (**Figure 3**, **Table 3**). Splicing mutations can result in complete exon skipping, intron retention, or the introduction of a new splice site within an exon or intron (72). However, mutations that do not destroy or create splice sites can also activate preexisting pseudosplice sites. These variants can also affect the fine balance of isoforms arising from alternatively spliced exons and thus cause disease.

**Table 3:** Mutations of splice or associated with splicing have been reported in IBD of children and adults.

Gene	Full name	Gene annotation	Locus	Mutation site	Potential function	Disease	Adults/Children	Ref.
CARD9	Caspase recruitment domain family member 9	It encodes a member of the caspase-associated recruitment domain (CARD) protein family.	9q34.3	c.1434+1G>C	Affected IBD risk	IBD	Adults	(41–43)
CUL2	Cullin 2	It enables ubiquitin protein ligase binding activity.	10p11.21	c.1675-5T>C	Affected IBD risk	IBD	Adults	(41)
IL12RB1	Interleukin-12 receptor subunit beta 1	It encodes a type I transmembrane protein belonging to the hemopoietin receptor superfamily that is part of the IL12 receptor complex.	19p13.11	c.1791+2T>G	Pathogenesis	CD	Adults	(44)
				c.684C>T	Diagnostic and therapeutic target	VEO-IBD	Children	(45)
MSH2	MutS homolog 2	It is homologous to the *E. coli* MutS gene and is involved in DNA mismatch repair (MMR).	2p22-p21	c.2006-6T>C	Excess risk of colorectal cancer for patients with UC	UC	Adults	(46)
NCF4	Neutrophil cytosolic factor 4	It encodes the p40-phox subunit of nicotinamide adenine dinucleotide phosphate (NADPH) oxidase.	22q13.1	c.32+1258T>C	Pathogenesis	CD, VEO-IBD	Adults and children	(47–49)
NOD2	Nucleotide-binding oligomerization domain containing 2	It encodes a protein with two CARD domains and six leucine-rich repeats (LRRs).	16q21	c.74-7T>A	Pathogenesis	IBD1, EO-IBD	Adults and children	(50–52)
				c.2798+158C>T	Affected CD (IBD1) risk	IBD1	Adults	(53, 54)
				c.2798+158C>T	Pathogenesis	IBD1, EO-IBD	children	(52)
				c.2462+7G>T	Pathogenesis	IBD1	children	(55)
ANKZF1	Ankyrin repeat and zinc finger domain containing 1	It is predicted to enable metal ion binding activity and it is involved in cellular response to hydrogen peroxide.	2q35	c.262-97G>A	Pathogenesis	Infantile- onset-IBD	children	(56)
CD40LG	CD40 ligand	It encodes a transmembrane molecule, CD40 ligand, found on T cells.	Xq26	c.410-2A>T	Impacted management	VEO-IBD, EO-IBD	children	(57)
CTLA4	Cytotoxic T-lymphocyte–associated protein 4	It encodes a protein that transmits an inhibitory signal to T cells and is a member of the immunoglobulin superfamily.	2q33	c.457+1G>A	Impacted treatment	Pediatric IBD	children	(58)
IL10RA	Interleukin-10 receptor subunit alpha	It encodes a receptor for interleukin-10.	11q23	c.188+1G>A	Pluripotency markers	IBD28	children	(59)
				c.537G>A	Pathogenesis	IBD28		(32, 60–64)
				c.368-10C > G	Pathogenesis	VEO-IBD (IBD28)		(65)
IL10RB	Interleukin-10 receptor subunit beta	It encodes a family of cytokine receptors that are accessory chains necessary for active interleukin-10 receptor complexes.	21q22.11	c.646+2T>C	Pathogenesis	IBD25	children	(66)
SKIV2L	Ski2 like RNA helicase	It encodes DEAD box proteins that is characterized by the conserved motif Asp-Glu-Ala-Asp (DEAD), are putative RNA helicases.	6p21	c.354+5G>A	Pathogenesis	UC	children	(67)
				c.355-2A>C	Pathogenesis	VEO-IBD		(68)
STXBP3	Syntaxin binding protein 3	It activates syntaxin binding activity and is involved in the negative regulation of neutrophil degranulation, calcium-dependent exocytosis and platelet aggregation.	1p13.3	c.1029+1G>A	Pathogenesis	VEO-IBD	children	(69)
TTC7A	Tetratricopeptide repeat domain 7A	It encodes a protein containing tetratricopeptide repeats.	2p21	c.844-1G>T	Pathogenesis	VEO-IBD	children	(70)
				c.1204-2A>G	Pathogenesis			(70)
TTC37	Tetratricopeptide repeat domain 37	It encodes a protein with 20 tetratricopeptide (TPR) repeats.	5q15	c.4497-1G>A	Impacted management of patients	VEO-IBD, EO-IBD	children	(57)
WAS	Wiskott–Aldrich syndrome	The Wiskott–Aldrich syndrome (WAS) protein family is involved in the transduction of signals from receptors on the cell surface to the actin cytoskeleton.	Xp11.4-p11.21	c.463+8G>A	Pathogenesis	EO-IBD	children	(66)
				c.360 + 1G > C	Pathogenesis	VEO-IBD		(71)

VEO-IBD, very early–onset IBD; EO-IBD, early-onset IBD.

### 4.1 Mutations of splicing in adults with IBD

Caspase recruitment domain family member 9 (*CARD9*), located on chromosome 9q34.3, is a susceptibility gene for IBD that encodes a connexin that integrates signals downstream from pattern recognition receptors and plays a key role in immune responses against microbes (73). To date, it has been reported that a splicing variant of *CARD9* (c.1434+1G>C) may affect IBD risk in adults (41–43). *CARD9* is involved in the nucleotide-binding domain (NOD)–like receptor signaling pathway, which is involved in the occurrence of IBD (**Figure 2**) (74). Except for *CARD9*, there are many susceptibility genes for IBD (75). However, only some of them with splicing mutations are associated with IBD. Meanwhile, cullin 2 (*CUL2*), located on chromosome 10p11.21, is a protein coding gene, and its splicing site (c.1675-5T>C) has also been reported to affect IBD risk (42).

Furthermore, interleukin-12 receptor subunit beta 1 (*IL12RB1*), located on chromosome 19p13.11, has a 3′ splicing site mutation (c.1791+2T>G) that has been reported to be associated with pathogenesis of adults with CD (44). *IL12RB1* is involved in JAK-STAT signaling pathway in both Th1 and Th17 lymphocytes, leading to CD (**Figure 2**) (76). MutS homolog 2 (*MSH2*), located on chromosome 2p21-p16.3, has a splicing variant (c.2006-6T>C) that has been reported to be associated with an excess risk of colorectal cancer for patients with UC (46). Neutrophil cytosolic factor 4 (*NCF4*), located on chromosome 22q12.3, encodes the p40phox protein and is another susceptibility gene for IBD (77, 78). The splicing variant of *NCF4* (c.32+1258T>C) has been reported to be involved in the pathogenesis of CD (47, 48).

Moreover, nucleotide-binding oligomerization domain containing 2 (*NOD2*, OMIM #605956), located on chromosome 16q12.1, is associated with CD (IBD1, OMIM #266600) (53). NOD2 signaling in macrophages is strongly associated with increased bacterial susceptibility in CD (79). *NOD2* is involved in the signaling pathway in both antigen-presenting cells (APCs) and macrophages of CD (**Figure 2**) (33, 76, 80, 81). A splicing variant of *NOD2* (c.2798+158C>T) has been reported to affect CD risk (53, 54). Another splice site of *NOD2* (c.74-7T>A) may be associated with the pathogenesis of CD (50, 51). In total, seven splicing mutations from six genes (*CARD9*, *CUL2*, *IL12RB1*, *MSH2*, *NCF4*, and *NOD2*) have been reported to be related to IBD (including CD and UC) in adults (**Table 3**). They may be involved in pathogenesis or influenced disease risk. *NOD2* is directly related to the genetic typing of IBD (IBD1), and further studies on these splicing sites will be of great help to the pathogenesis, diagnosis, and treatment of IBD1.

### 4.2 Mutations of splicing or associated with splicing in children with IBD

**Figure 4:**
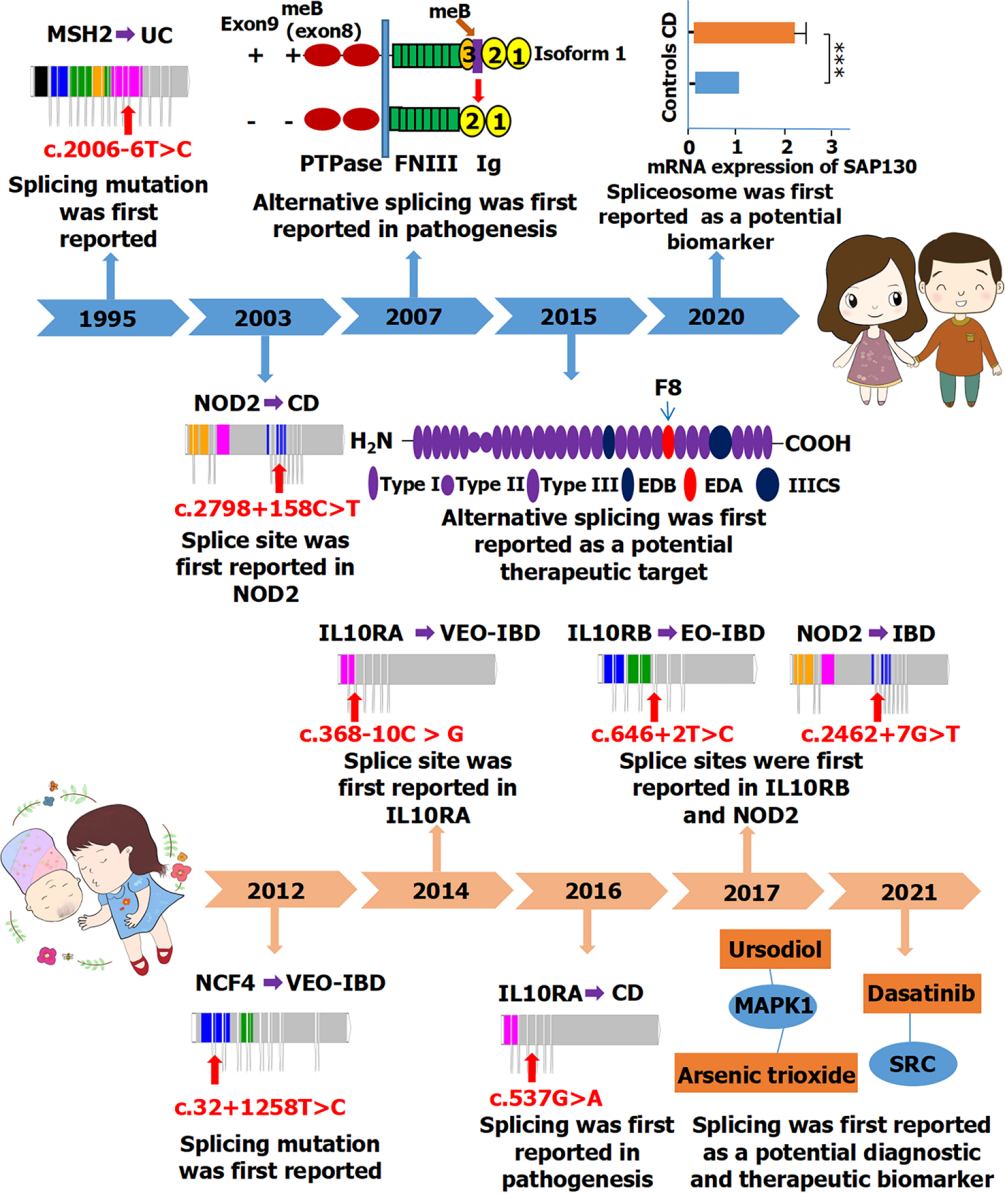
A timeline of alternative splicing and splicing mutations reported in IBD (including CD and UC) between adults (Top) and children (Bottom). PTPase, protein-tyrosine phosphatase; FNIII, fibronectin type III; SAP130, spliceosome-associated protein 130; ***P < 0.001; IIICS, type III connecting segment; MAPK1, mitogen-activated protein kinase 1; SRC, proto-oncogene tyrosine-protein kinase Src. The full names of those genes are shown in **Table 3**.

Ankyrin repeat and zinc finger domain containing 1 (*ANKZF1*), located on chromosome 2q35, is associated with infantile-onset IBD (56). The splicing variant of *ANKZF1* (c.262-97G>A) has been reported to be involved in the pathogenesis of infantile-onset IBD (56). In their study, four infantile-onset patients with IBD carried *ANKZF1* mutations on one or two alleles. When only IBD with onset before 6 months of age was considered, the mutation frequency of *ANKZF1* in their study population was 100%, indicating that *ANKZF1* is indeed important in the pathogenesis of infantile-onset IBD. CD40 ligand (*CD40LG*), located on chromosome Xq26, is associated with VEO-IBD and EO-IBD (57). The splicing variant of *CD40LG* (c.410-2A>T) may affect the treatment of VEO-IBD and EO-IBD (58). Meanwhile, cytotoxic T-lymphocyte associated–protein 4 (*CTLA4*), located on chromosome 2q33, is associated with pediatric IBD (58). The splicing variant of *CTLA4* (c.457+1G>A) may affect the treatment of pediatric IBD through regulatory T cells and immune modulation (58). Whereas *IL12RB1* is associated with CD in adults, it is also associated with IBD in children (45). The variant of *IL12RB1* (c.684C>T) plays a very important role in the splicing process and might be a diagnostic and therapeutic target for VEO-IBD (45). Furthermore, another gene, *NCF4*, is associated with CD in adults and with IBD in children. Another splicing variant of *NCF4* (c.32+1258T>C) may be involved in the pathogenesis of VEO-IBD (**Figure 4**) (49).

In addition, ski2-like RNA helicase (*SKIV2L*), located on chromosome 6p21, is associated with VEO-IBD and UC in children (67, 68). Whereas the splicing variant of *SKIV2L* (c.354+5G>A) may be associated with the pathogenesis of UC in children, another splicing variant (c.355-2A>C) may be involved in the pathogenesis of VEO-IBD (67, 68). Syntaxin binding protein 3 (*STXBP3*), located on chromosome 1p13.3, is associated with VEO-IBD (69). A splicing variant of *STXBP3* (c.1029+1G>A) may be associated with the pathogenesis of VEO-IBD (70). Tetratricopeptide repeat domain 7A (*TTC7A*), located on chromosome 2p21, is also associated with VEO-IBD (70). Two splicing variants of *TTC7A* (c.844-1G>T and c.1204-2A>G) may be involved in the pathogenesis of VEO-IBD (70). Tetratricopeptide repeat domain 37 (*TTC37*), located on chromosome 5q15, is associated with VEO-IBD and EO-IBD (57). A splicing variant of *TTC37* (c.4497-1G>A) may affect the treatment of VEO-IBD and EO-IBD (57). Wiskott–Aldrich syndrome (*WAS*), located on chromosome Xp11.4-p11.21, is associated with EO-IBD and VEO-IBD (66, 71). Splicing variants of *WAS* (c.463+8G>A and c.360+1G>C) may be involved in the pathogenesis of EO-IBD and VEO-IBD, respectively (66, 71).

Moreover, whereas *NOD2* is associated with CD (IBD1) in adults, it is also associated with IBD1 in children (52, 71). Three splicing variants of *NOD2* (c.74-7T>A, c.2798+158C>T, and c.2462+7G>T) may be involved in the pathogenesis of IBD1 in children (52, 55). Interleukin-10 receptor subunit beta (*IL10RB*, OMIM #123889), located on chromosome 21q22.11, is associated with IBD25 (OMIM #612567) (82). A splicing variant of *IL10RB* (c.646+2T>C) may be involved in the pathogenesis of EO-IBD (IBD25) (66). Interleukin-10 receptor subunit alpha (*IL10RA*, OMIM #146933), located on chromosome 11q23, is associated with IBD28 (OMIM #613148) (59). Splicing variant of *IL10RA* (c.188+1G>A) may be a pluripotency marker for IBD28 (59). A synonymous variant in *IL10RA* (c.537G>A) may be associated with the pathogenesis of IBD28 by affecting RNA splicing (32, 60–64). Another splicing variant of *IL10RA* (c.368-10C>G) may also be associated with the pathogenesis of VEO-IBD (IBD28) (65). Both *IL10RA* and *IL10RB* are involved in the JAK-STAT signaling pathway in Th2 lymphocytes, leading to UC (**Figure 2**) (76).

In the signaling pathway, some genes are involved in the NOD-like receptor signaling pathway, Toll-like receptor signaling pathway, and JAK-STAT signaling pathway connected with CD, including *CARD9*, *NOD2*, and *IL12RB1*, whereas *IL10RA* and *IL10RB* are involved in the JAK-STAT signaling pathway connected with UC (**Figure 2**). Compared with adults, more mutations and genes have been reported in children. This may be attributed to the fact that patients with monogenic IBD before the age of 6 years account for the majority of cases (63.4%), whereas adolescent and adult-onset monogenic IBD account for one-third of cases (83). Approximately, 20 splicing mutations in 13 genes (*ANKZF1*, *CD40LG, CTLA4, IL10RA*, *IL10RB*, *IL12RB1*, *NCF4*, *NOD2*, *SKIV2L*, *STXBP3*, *TTC37*, *TTC7A*, and *WAS*) have been reported to be associated with IBD in children (**Table 3**). Among them, splicing mutations of *IL12RB1*, *NCF4*, and *NOD2* have been reported both in adults and pediatric IBD. Some may play a role in pathogenesis, whereas others may act as markers or affect treatment. Some may even serve as diagnostic or therapeutic targets. In children, there were two synonymous variants that affected RNA splicing, which could participate in pathogenesis or serve as diagnostic or therapeutic targets.

## 5 Conclusions and future perspectives

Alternative splicing may be related to pathogenesis, biomarker for diagnosis, and therapeutic targets for both adults and children with IBD. The study of alternative splicing has been reported more in adults, but children have more splicing mutations and genes associated with IBD. Splicing was first reported in adults with IBD in 1995, whereas studies on splicing in children with IBD only began in the last decade (**Figure 4**). Some splicing factors have been reported to be involved in regulating alternative splicing events in adult patients with IBD. Thus far, no related splicing factors have been reported in children; therefore, research should be strengthened in this regard.

A total of 24 mutations of splicing or associated with splicing in 16 genes have been reported to be associated with IBD in children or adults. Whereas seven splicing mutations from six genes have been reported to be related with IBD (including CD and UC) in adults, 20 splicing mutations in 13 genes have been reported to be associated with IBD in children. Splicing mutation sites and genes are much more frequently reported in children. Importantly, three of these genes are associated with IBD1, IBD25, and IBD28, including *NOD2*, *IL10RB*, and *IL10RA*, respectively.

Nevertheless, there are no validated splicing events that have a causative component of IBD. There are no validated splicing events that are just a consequence of IBD. No splicing events are employed routinely in the therapy of IBD. There are no validated or approved biomarkers of IBD that employ splicing events and are used by clinicians. No splicing-related events are employed routinely for the direct benefit of the patient with IBD. However, studies have shown that some alternative splicing may serve as potential biomarkers or therapeutic targets for IBD (**Table 1**) (18–20, 23, 25–27, 31). We suggest that the study of alternative splicing in IBD can be strengthened in the future, especially in the study of three genes (*NOD2*, *IL10RB*, and *IL10RA*). At present, research on alternative splicing in IBD is still limited. Future studies are needed to understand the role of alternative splicing in IBD and to find new pathogenesis, diagnostic, and therapeutic targets for children and adults with IBD. If biomarkers and therapeutic targets for IBD can be found in this field, then it will provide convenience for the diagnosis and treatment of IBD.

## Author contributions

JZ, QZ, YZ, YS, YL, MC, SZ, and ZW designed and drafted the article. MC, SZ, and ZW reviewed and revised the manuscript. All authors contributed to the article and approved the submitted version.

## Supplementary material

Supplementary Table 1 Bibliometric analysis of the studies on alternative splicing and inflammatory bowel disease (IBD) (including Crohn’s disease (CD) and ulcerative colitis (UC)) based on the web of science core collection database. A. Histogram with Alternative splicing, IBD (including CD and UC) as keywords for the last decade (2012-2021), based on the Web of Science core collection database. Approximately 6-19 articles have been published in this category each year for the last decade. B. Heatmap of bibliometric analysis, the number of documents with “INFLAMMATORY-BOWEL-DISEASE” “ULCERATIVE-COLITIS” “CROHNS-DISEASE” “EXPRESSION” “ACTIVATION” “GENOME-WIDE ASSOCIATION” “PATHOGENESIS” “MECHANISMS” “SPLICE VARIANT” “SPLICE-SITE MUTATION” as the keywords in the Web of Science core collection database. Color saturation indicates the strength of citation keyword. C. Network diagram for bibliometric analysis of alternative splicing in studies of IBD (including CD and UC) based on the Web of Science core collection database. Nodes represent unique keywords; node size is proportional to the number of references; node colors indicate modules. Some keywords are related together, such as Ulcerative colitis, splicing variant and colorectal cancer, while the remaining keywords are related in other three parts. Note: NF kappa B, nuclear factor- kappa B; ER stress, endoplasmic reticulum stress.
