# Supplementary figures and images for: The regulatory role of alternative splicing in inflammatory bowel disease
Source: Front Immunol. 2023 Apr 21;14:1095267. doi: 10.3389/fimmu.2023.1095267 (PMC10160418; doi:10.3389/fimmu.2023.1095267)

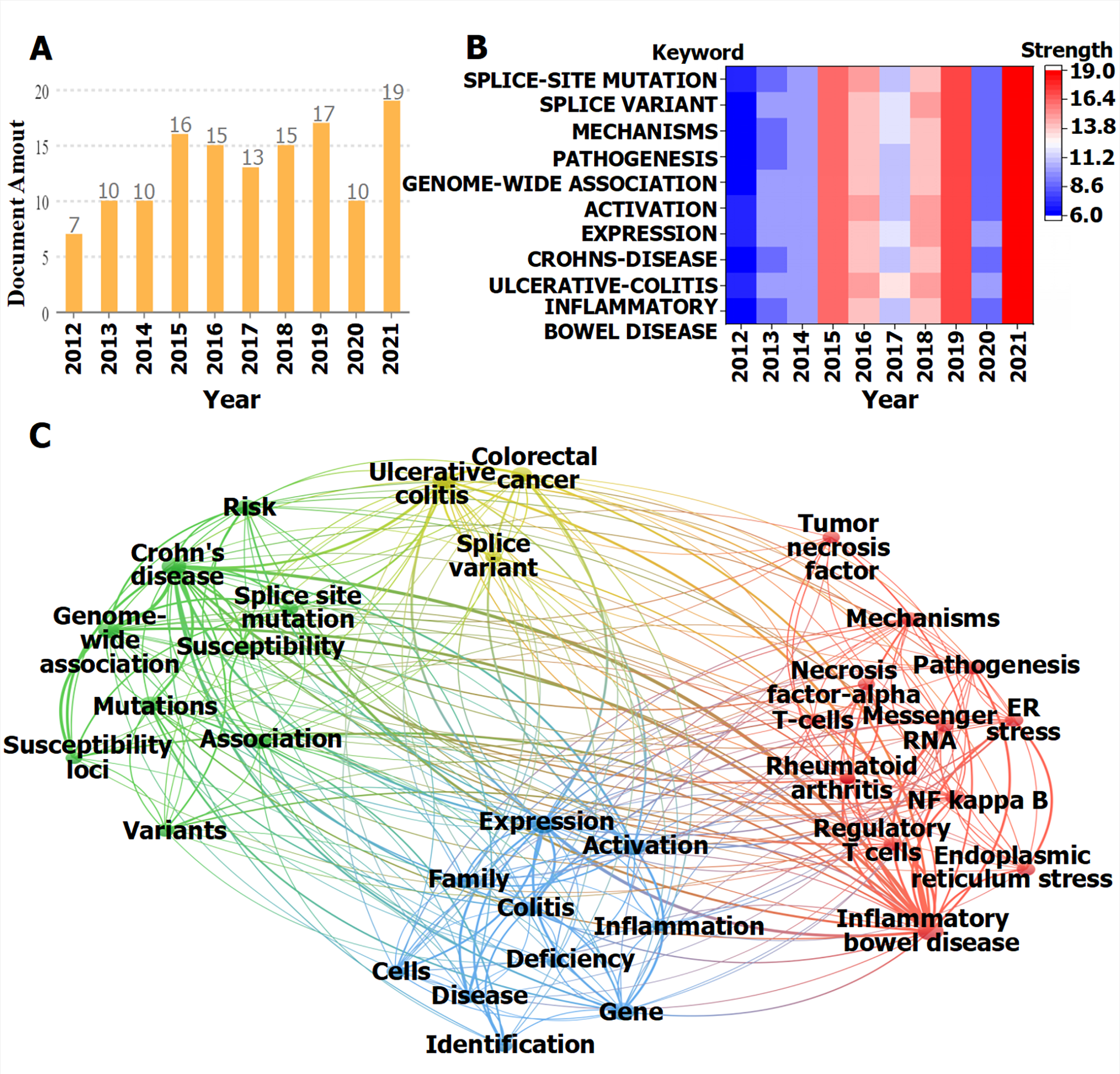

Supplement: Supplementary file 2 [file Image_1.tif]
